# Biochemical parameters in diabetic pregnant rats treated with Bauhinia holophylla aqueous extract

**DOI:** 10.1186/1758-5996-7-S1-A78

**Published:** 2015-11-11

**Authors:** Thais Leal Silva, Kamirri Savazzi, Jeferson José Silva Sousa, Rafaianne Queiroz de Moraes Souza, Thaigra de Sousa Soares, Marcelo Salvatte Pinheiro, Kleber Eduardo de Campos, Gustavo Tadeu Volpato

**Affiliations:** 1 Universidade Federal de Mato Grosso, Barra do Garças, Brazil

## Background

Several medicine plants are used by population around the world for diabetic treatment. Bauhinia holophylla, commonly known as paw-of-cow, is a plant used to diabetes treatment. However, there are no studies to prove the effects of this plant.

## Objective

To evaluate the effect of B. holophylla aqueous extract treatment on biochemical parameters in blood and oxidative stress in diabetic and non-diabetic pregnant rats.

## Materials and methods

Diabetes was induced by streptozotocin (40 mg/kg) in virgin female Wistar rats. After diabetes status confirmation, rats were mated. The pregnant diabetic rats were divided in four experimental groups (n minimum=12 animals/group): Non-diabetic; Non-diabetic Treated; Diabetic and Diabetic Treated. Oral administration of leaves aqueous extract of Bauhinia holophylla was given to non-diabetic and diabetic pregnant rats at increasing doses: 200mg/kg between day 0 and 7 of pregnancy, 400 mg/kg between day 8 and 14 and 800 mg/kg between day 15 and 21. On day 21 of pregnancy, all rats were anesthetized and killed, and the blood and liver were collected. The biochemical serum parameters (glycemic level, alanine aminotransferase [ALT], protein, cholesterol, triglycerides, High-density level lipoprotein [HDL]) and hepatic oxidative stress biomarkers (malondialdehyde [MDA], superoxide dismutase [SOD], catalase, total glutathione, thiol group) were analyze. Analysis of variance followed by Tukey's test was used. Differences were considered statistically significant when p<0.05.

## Results

Non-diabetic and diabetic rats presented no glycemic changes. All the experimental groups showed decrease values in HDL levels compared to control group. Both diabetic groups showed higher levels of triglycerides, ALT, and MDA, and decreasing levels in catalase. Moreover, the treatment with B. holophylla in diabetic group decreased triglycerides and cholesterol levels and increased HDL compared to diabetic non-treated animals.

## Conclusion

The aqueous extract from B. holophylla leaves failed to modify the maternal hyperglycemia and stress oxidative. However, this plant improves the biochemical parameters.

